# Simultaneous Quantification and Pharmacokinetic Study of Five Homologs of Dalbavancin in Rat Plasma Using UHPLC-MS/MS

**DOI:** 10.3390/molecules25184100

**Published:** 2020-09-08

**Authors:** Difeng Zhu, Li Ping, Yawen Hong, Jiale Shen, Qinjie Weng, Qiaojun He

**Affiliations:** Center for Drug Safety Evaluation and Research, College of Pharmaceutical Sciences, Zhejiang University, Hangzhou 310058, China; zhudf@zju.edu.cn (D.Z.); pingli552@zju.edu.cn (L.P.); hongyawen61@zju.edu.cn (Y.H.); shenjiale@zju.edu.cn (J.S.)

**Keywords:** dalbavancin, homolog, isomer, UHPLC-MS/MS, rat plasma, pharmacokinetics study

## Abstract

Dalbavancin is a novel semisynthetic glycopeptide antibiotic that comprises multiple homologs and isomers of similar polarities. However, pharmacokinetic studies have only analyzed the primary components of dalbavancin, namely B_0_ and B_1_. In this study, an ultra-high-performance liquid chromatography-tandem mass spectrometry (UHPLC-MS/MS) method was developed to simultaneously determinate and investigate the five homologous components of dalbavancin, namely, A_0_, A_1_, B_0_, B_1_, and B_2_, in rat plasma. In this method, methanol was used to precipitate plasma, and a triple-bonded alkyl chromatographic column was used for molecule separation, using 0.1% formic acid-acetonitrile as the mobile phase for gradient elution. Targeted homologs were analyzed by a triple quadrupole mass spectrometer using positive electrospray ionization in multiple reaction monitoring mode. The linearity range was 50–2500 ng/mL with a high correlation coefficient (*r*^2^ > 0.998). This method was successfully applied in the pharmacokinetic analysis of dalbavancin hydrochloride to investigate dalbavancin components in rats.

## 1. Introduction

Methicillin-resistant *Staphylococcus aureus* (MRSA) is the primary pathogen associated with hospital- and community-acquired infections [1], which are mitigated using glycopeptide antibiotics [2]. First-generation glycopeptide antibiotics, developed in the 1950s, are directly derived from metabolites of microorganisms. These antibiotics have a strong efficacy against drug-resistant infections caused by gram-positive bacteria, especially MRSA. However, the extensive use of these antibiotics has led to a gradual reduction in the sensitivity of MRSA to these drugs, resulting in the emergence of drug-resistant strains [3].

**Figure 1:**
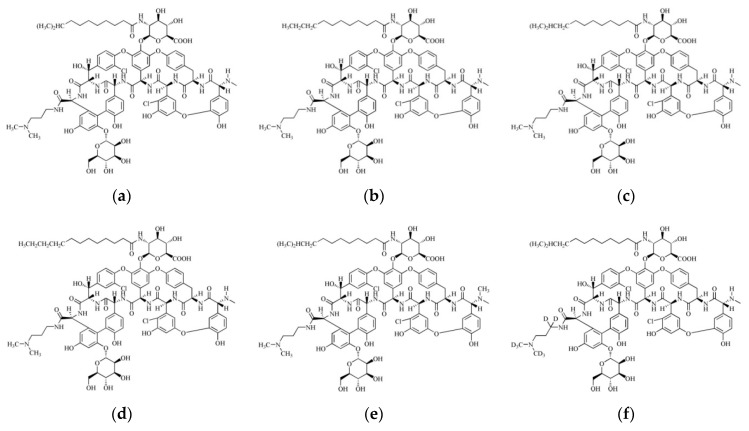
Structural representation of the components of dalbavancin and D_8_-labeled internal standard (IS): (**a**) A_0_, (**b**) A_1_, (**c**) B_0_, (**d**) B_1_, € B_2_, (**f**) D_8_-labeled IS.

Dalbavancin (formerly known as MDL-63397, A-A1, and BI-397 [4]), a novel semisynthetic glycopeptide antibiotic developed by Durata Therapeutics, was first approved for clinical use in the United States in 2014, primarily to treat acute bacterial skin infections caused by gram-positive bacteria in adults [5]. Its spectrum includes MRSA, methicillin-sensitive *Staphylococcus aureus*, coagulase-negative staphylococci, and streptococci. Dalbavancin has demonstrated antibacterial activity equivalent or stronger to that of vancomycin and teicoplanin [6]. Dalbavancin is derived from the natural glycopeptide antibiotic, A40926, through amidation of the terminal carboxy group and is composed of five components, A_0_, A_1_, B_0_, B_1_, and B_2_, sharing the same parent nucleus (Figure 1) [7] with B_0_ being the highest proportion (> 75%) [8] and the primary active substance. Thus, pharmacokinetic studies have primarily focused on B_0_ (or B_0_ and B_1_). The activity of dalbavancin is primarily based on the unique pharmacokinetic properties of this component, which has a long half-life and can be administered weekly [9,10,11].

The pharmacokinetics of B_0_/B_1_ have been thoroughly studied, while that of the remaining components have not. Phase II and III clinical trials have reported various adverse events (AE) of dalbavancin including nausea (5.5%), headache (4.7%), and diarrhea (4.4%) [12]. It remains unclear whether these Aes are associated with some or all components of dalbavancin; hence, it is important to study their pharmacokinetics. Among the common detection methods used for multi-component antibiotic samples of biological origin, ELISA [13,14], drug-derived radioactivity [15,16], LC [17,18], LC-MS [19,20], and CE [21,22] have proven effectiveness. However, the composition of dalbavancin is more complex; besides being homologs, A_0_ is an isomer of A_1_, while B_0_ is an isomer of B_1_. The only difference between B_0_ and B_2_ is a methyl group at the amino side chain terminal; however, the polarities of the components are very similar. This makes detection and quantification of these components using simple mass spectrometry and liquid chromatography challenging.

Table 1 lists various analytical methods used for the detection and quantification of dalbavancin in biological samples. Among all reported methods, most lower limit of quantification (LLOQ) values of dalbavancin plasma samples were ≥ 0.5 μg/mL. Although there is one report on UHPLC-MS/MS with lower LLOQ, it is only suitable for the determination of B_0_/B_1_ with a narrow linear region. In addition, A_0_, A_1_, and B_2_ are present at very low concentrations. The concentrations of several dalbavancin components in plasma samples are < 0.1 μg/mL at the terminal stage of elimination. These analytical methods are not adequate for pharmacokinetic studies of various dalbavancin components. Therefore, it is necessary to develop a highly sensitive and selective method to simultaneously quantify the components of dalbavancin to accurately study and understand the pharmacokinetic characteristics of each component.

In this study, a UHPLC-MS/MS method was established to determine the concentration of various components of dalbavancin in rat plasma, thus laying the foundation for subsequent studies in humans. To the best of our knowledge, this is the first study of its kind using this method to determine the concentration of various dalbavancin components.

## 2. Results and Discussion

### 2.1. Optimization of the UHPLC-MS/MS

The optimization results of compounds were obtained using scanning mass spectrometry in the positive ion (ESI+) and negative ion (ESI−) modes. Owing to the multiple acidic and basic ionizable groups of these molecules, they could easily form species with two positive charges (H^+^) during ionization. The precursor ion of each component was determined and optimized using cone voltage and collision energy, respectively. By examining the D_8_-labeled internal standard (IS) of all dalbavancin components and component B_0_, we determined that the signal intensity could be further improved by promoting the formation of [M+2H]^2+^ adducts. Therefore, [M+2H]^2+^ adducts were selected as precursor ions and the positive ion (ESI+) mode was adopted for detection. To determine the best product ions for the selected reaction monitoring (SRM) transition of these adducts, one optimization quantitative product ion was selected from three different candidate fragments for each precursor and the MS detection conditions were preliminarily obtained. To obtain stable and highly sensitive IS, more stable deuterated D_8_-labeled IS was selected. Next, optimal mass-spectrometry conditions for each component and D_8_-labeled IS were further optimized, including the cone voltage and collision energy, as well as the precursor and product ions. Lastly, various parameters were obtained and applied to the final MS method (Table 2).

However, several components in dalbavancin are homologs, while others are isomers. During mass spectrometry, there are several components with the same mass-to-charge ratio (*m*/*z*) of precursor and product ions, which cannot be accurately separated using a single mass spectrometry analysis. Therefore, LC separation has become the primary method. Various UHPLC columns with different particle sizes, pore diameters, and column lengths have been investigated. Based on optimized experiments, an ACQUITY UHPLC BEH C18 (2.1 mm × 100 mm, 1.7 μm) triple-bonded alkyl column was selected. Peak patterns of compounds could be obtained using this chromatographic column, and eventually, other chromatographic columns were eliminated. Simultaneously, several elution methods were selected (including methanol, with acetonitrile as the organic modifier, and formic acid water, with ammonium formate water at different concentrations as additives). It was found that the separation effect of the analytes could be significantly improved by using acetonitrile as the organic phase and adding 0.1% (*v*/*v*) formic acid as an additive. In particular, the effective separation of these compounds was observed using an organic phase comprising 30–33% acetonitrile (the elution time was 1–6 min). The retention times of dalbavancin A_0_, A_1_, B_0_, B_1_, and B_2_ were 3.20, 3.42, 4.69, 5.03, and 4.92 min, respectively (Table 2). Finally, 0.1% formic acid water (A) and acetonitrile (B) were selected as the mobile phases for gradient elution.

### 2.2. Optimization of Sample Preparation

Studies have reported various methods for biological sample extraction, such as protein precipitation [34], enzymatic hydrolysis [35], and extraction [36]. Considering the low in vivo concentrations of some target substances (including A_0_, and A_1_) and the need for relatively low LLOQ, a good extraction method should eliminate interferences caused by impurities, so that the extraction recovery rate of each component can be within the acceptable range. Other studies have reported the use of acetonitrile precipitation extraction as a sample pretreatment method [23,37]. While the recovery rates of B_0_ and B_2_ were both between 70% and 100%, the recovery rates of other components were not high. Therefore, methanol precipitation and methanol plus acid precipitation were investigated based on drug properties. Subsequently, methanol precipitation was selected and a good extraction recovery rate was achieved.

### 2.3. Method Validation

All dalbavancin components showed good linearity in the range of 50 to 2500 ng/mL. The verification contents of all dalbavancin components were acceptable, and the intra- and inter-batch precision and accuracy at low, medium, and high concentrations were within the acceptable ranges [38].

#### 2.3.1. Specificity, Linearity, Limit of Detection (LOD), and LLOQ

**Figure 2:**
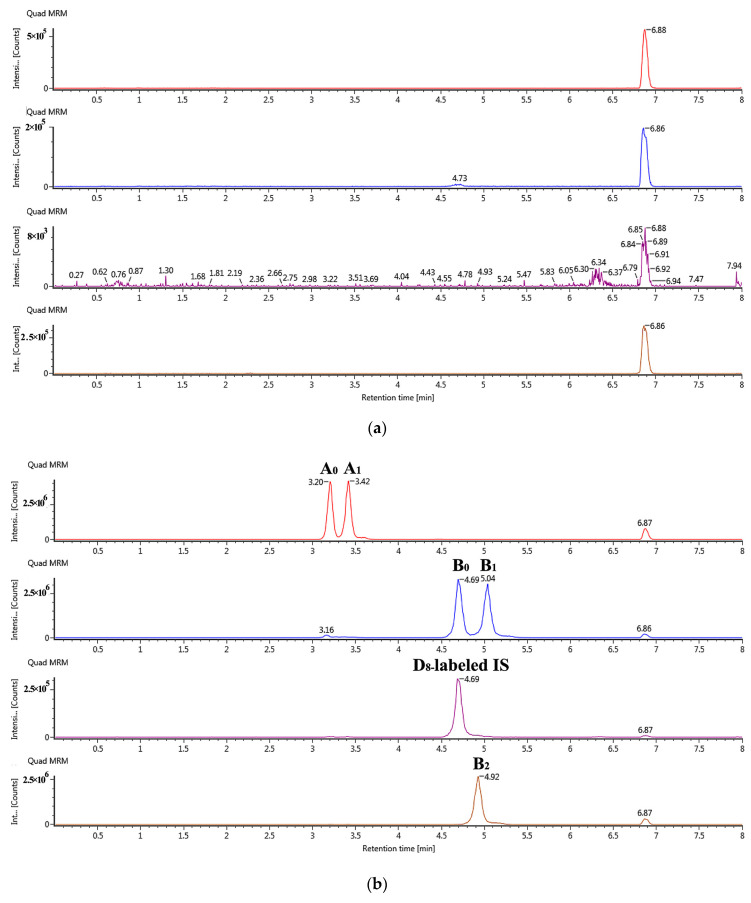

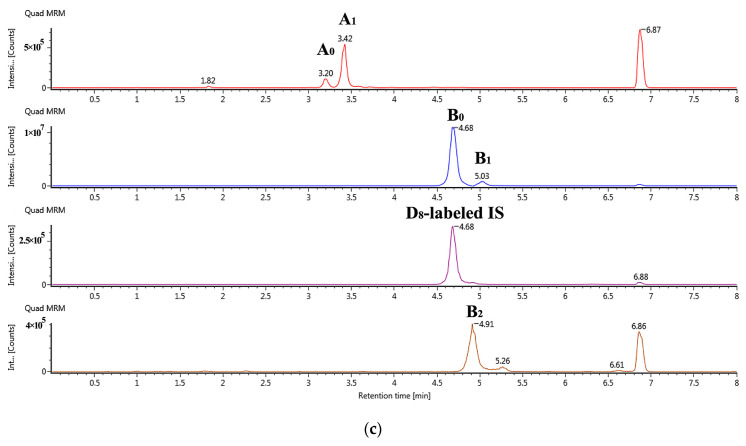
Selected ion chromatograms for LC-MS/MS determination of dalbavancin components in rat plasma: (**a**) blank biological sample, (**b**) upper limit of quantification: 2000 ng/mL, (**c**) the plasma sample 3 h following the administration spike with IS.

Qualified specificity indicates that the analysis method can distinguish target compounds from other interfering substances. The chromatogram obtained (Figure 2) showed that no interference signal was detected within the retention time ± 2.5% of all target components during the analysis process of blank biological samples. In addition, all components were well separated, indicating that the method had good specificity.

The linearity of the predetermined range (50, 100, 250, 500, 1250, 2000, and 2500 ng/mL) was evaluated by measuring the correlation coefficient *r*^2^ using the fitted standard curve. The correlation coefficient of each component was above 0.998, suggesting good linearity.

Table 3 summarizes the LOD and LLOQ of each component using this detection method. The LOD values of these components ranged from 5 ng/mL to 10 ng/mL, thus meeting the drug concentration determination limits of dalbavancin in vivo.

#### 2.3.2. Recovery, Matrix Effect, Intra-Day Precision, and Inter-Day Precision

According to the guidelines of Commission Regulation (EU) 2006/401 [39], the recovery rates (71.2–90.2%) and matrix effect (ME) values (97.5–101.7%) of all dalbavancin components were within acceptable ranges. The results of intra-day precision and inter-day precision are summarized in Table 4. All values were < 10%.

#### 2.3.3. Stability, Dilution Reliability, and Residue Verification

The stock solution of dalbavancin components and those of deuterated dalbavancin post freezing (−16 °C to −24 °C) for 7 days had a change rate of −4.39% to 3.76%, indicating good stability. Storage of whole blood samples at room temperature (20 °C to 24 °C) for 12 h, plasma samples on ice for 4 h, plasma samples after treatment in an automatic sampler for 10 h, plasma samples after treatment at room temperature for 4 h, three freeze-thaw cycles, and storage at −16 °C to −24 °C for 28 days all showed good stability. The results showed that all dalbavancin components were stable under the above conditions.

The accuracy and precision of B_0_, B_1_, A_1_, and B_2_ during the plasma sample determination process under the corresponding dilution factors were within the ± 15% range, indicating that plasma samples with dalbavancin B_0_, B_1_, A_1_, and B_2_ were stable under 50× dilution, and dalbavancin B_0_ plasma samples were stable under 1000× dilution. The residue was determined by measuring the blank plasma sample after detecting the upper limit of the standard curve, and the residual response measured in the blank sample was not more than 20% of the LLOQ, indicating that the residue met the requirements.

### 2.4. Application to Pharmacokinetic Study in Rats

A total of 20 animal plasma samples were analyzed using the verified UHPLC-MS/MS detection method to evaluate 28-day multiple-dose pharmacokinetics of dalbavancin hydrochloride injected daily in rats. Simultaneously, incurred sample reanalysis showed that the difference between the test results and the original values was less than 20%, and the QC samples had passed the pre-established standard (at least two-thirds of the samples should have a concentration difference within ±20%). The results confirmed that the developed method could detect dalbavancin components in rat plasma.

**Figure 3:**
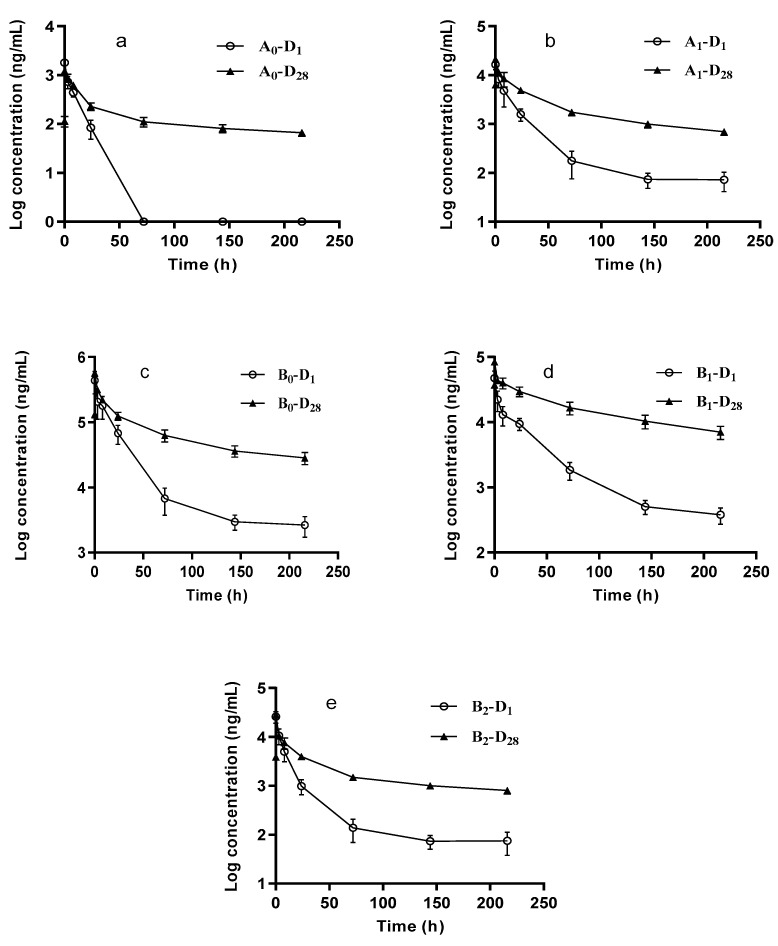
Semi-logarithmic plot of the dalbavancin components mean plasma concentration-time curves following IV administration of 40 mg/kg dalbavancin hydrochloride in rats: (**a**) A_0_, (**b**) A_1_, (**c**) B_0_, (**d**) B_1_, (**e**) B_2_.

The average drug concentration-time curves of dalbavancin components in rats following the administration of 40 mg/kg dalbavancin hydrochloride injection on days 1 and 28 are shown in Figure 3. The final calculation results are shown in Table 5. Results from the pharmacokinetics study showed that the plasma *AUC*_(0–∞)_ of component B_0_ was the highest after the first day of drug administration, which was approximately 5.8 times higher than the other components combined. After 28 days of continuous drug administration, the plasma *AUC*_(0–∞)_ values of dalbavancin components in vivo increased 2–6 times compared with those on the first day, suggesting that dalbavancin can accumulate. The *AUC*_(0–t)_ on day 28 (D_28_) was higher than that on day 1 (D_1_), further indicating that the drug had potentially accumulated; the concentration after the first day of treatment was equal to or lower than that of *C*_0_ on D_28_ (Figure 3). Furthermore, the elimination process slowed down, consequently prolonging the elimination half-life (*T*_1/2_). The effective *T*_1/2_ of dalbavancin components, except for component B_0,_ in vivo increased with the duration of drug administration (*p* < 0.05). These results suggested that accumulation of dalbavancin might be due to enzyme inhibition or saturable elimination. As the duration of drug administration increased, the *T*_1/2_ of components A_0_, A_1_, and B_2_ exceeded that of B_0_ and B_1_. During treatment, the accumulation risk caused by the increase in *T*_1/2_ of these components should be considered and their associated toxic effects further investigated.

## 3. Materials and Methods

### 3.1. Reagents and Materials

LC/MS grade methanol and acetonitrile were purchased from Merck KGaA (Darmstadt, Germany). Formic acid was purchased from Sigma-Aldrich Corp. (St. Louis, MO, USA), and Milli-Q water (18.2 MΩ cm) was prepared using a Purelab OptionS7 ultra-pure water system (ELGA LabWater, High Wycombe, UK). The components A_0_, A_1_, B_0_, B_1_, and B_2_ of dalbavancin, the reference substances, and the D_8_-labeled IS (Figure 1) were supplied by CTTQ Pharmaceutical group Co., Ltd. (Jiangsu, China), and the purities were 98.2, 97.0, 97.1, 98.3, 97.3, and 99.61%, respectively. Dalbavancin hydrochloride for injection (LOT: 701713F) was supplied by Durata Therapeutics, Inc. (Morristown, NJ, USA).

### 3.2. Standard Solutions

Dalbavancin (A_0_, A_1_, B_0_, B_1_, and B_2_) reference substances were prepared by dissolution in methanol:water (50:50, *v*/*v*) and diluted to 1 mg/mL stock solutions. The stock solutions of dalbavancin components and the deuterated dalbavancin stock solution were stored at −16 °C to −24 °C. The solutions were further diluted with methanol:water (50:50, *v*/*v*) to obtain the desired dalbavancin (A_0_, A_1_, B_0_, B_1_, and B_2_) standard curve working solutions.

### 3.3. Animal Experiments

All animal experiments were approved by the Animal Experiment Committee of the Center for Drug Safety Evaluation and Research of Zhejiang University. Sprague Dawley (SD) rats used in this study were purchased from Zhejiang Vital River Laboratory Animal Technologies Co., Ltd. (Zhejiang, China). The use of experimental animals followed the 3R (Reduction, Replacement, Refinement) principles. The animal experiment sites had passed the full accreditation of the Association for Assessment and Accreditation of Laboratory Animal Care International.

### 3.4. Sample Preparation

Blood samples were collected and centrifuged (5417R centrifuge, Eppendorf AG, Hamburg, Germany) at 835× *g* for 10 min to separate the upper plasma. Then, 50 μL of plasma was mixed with 200 μL of methanol (containing dalbavancin B_0_-D_8_ isotope internal standard solutions at a concentration of 100 ng/mL), after which the solution was swirled with a multi-tube vortex mixer (Allsheng, Hangzhou, China) for 10 min and centrifuged at 12,000× *g* for 10 min. To obtain a cleaner supernatant, it was further centrifuged at 12,000× *g* for 5 min before sample injection.

### 3.5. Liquid Chromatography-Tandem Mass Spectrometry

The UHPLC system consisted of an ACQUITY UPLC I-Class (Waters Corp., Milford, MA, USA) and separation was achieved on an ACQUITY UPLC BEH C18 (2.1 mm × 100 mm, 1.7 μm, Waters Corp.). The mobile phase contained 0.1% formic acid (phase A) and acetonitrile (phase B), while the gradient elution procedure was as follows; 0.00–1.00 min: 20%B→30%B, 1.00–6.00 min: 30%B→33%B, 6.01–7.00 min: 95%B, 7.00–7.01 min: 95%B→20%B, 7.01–8.00 min: 20%B. The flow rate was 0.300 mL/min, and the sample injection volume was 5.0 μL. The autosampler was maintained at 6 °C.

The mass spectrometry system used was Xevo TQ triple quadrupole MS/MS (Waters Corp.), equipped with ESI source. After injecting 500 ng/mL of dalbavancin component combined solution and dalbavancin isotope internal standard solution into the mass spectrometer, the optimized parameters of the instrument were obtained as follows: capillary voltage, 3.00 kV; ion source temperature, 150 °C; desolvation gas temperature, 350 °C; collision gas flow rate, 0.15 mL/min; desolvation gas flow rate, 650 L/h. SRM was used for monitoring the positive ion mode and the total analysis run time was 8 min. Data was processed using UNIFI v1.9.3 software (Waters Corp.).

### 3.6. Method Validation

Method validation was conducted primarily by reference to Commission Decision 2002/657/EC [40] and Commission Regulation (EU) 2006/401 [39]. The following items were investigated: specificity, linearity, precision and accuracy, reproducibility, LOQ, LOD, recovery rate, and ME.

#### 3.6.1. Specificity, Linearity, LOD, and LOQ

Specificity was investigated for the potential interferences by comparing three chromatograms of blank plasma of six SD rats, blank plasma-spiked five homologs and IS, and the plasma samples of dalbavancin 24 h following administration.

To evaluate linearity, blank SD rat plasma and dalbavancin were mixed, followed by the addition of standard solutions to prepare a series of simulated plasma working curves at the concentration range of 50–2500 ng/mL. Results were subjected to regression analysis, using the weighted (1/×) least square method, with the concentration of analyte as the abscissa and the peak area ratio of the analyte to the internal standard as the ordinate.

LOD was considered to be the lowest concentration of analyte detectable in the sample with a signal-to-noise (S/N) ratio > 3. LLOQ was considered to be the lowest concentration of analyte in a sample with a signal-to-noise (S/N) ratio > 10.

#### 3.6.2. Recovery and ME

To determine the recovery rate, blank SD rat plasma samples were mixed with different concentrations of QC solutions of dalbavancin components. Low-, medium- and high-concentration QC samples were prepared, with three samples for each concentration. The recovery rate was obtained by dividing the extracted sample response by the blank plasma spiked extract sample response values of the corresponding concentration.

For ME, blank plasma was taken from six SD rats and mixed with dalbavancin QC solutions at different concentrations, with six samples for each concentration. The ME was obtained by calculating the ratio between spiked mobile phase solutions and unextracted samples.

#### 3.6.3. Intra- and Inter-Day Precision and Accuracy

Blank SD rat plasma samples were mixed with dalbavancin component QC solutions (at different concentrations) in a centrifuge tube to prepare dalbavancin component plasma QC samples, with five samples for each concentration. Three batches were continuously detected to investigate the intra-batch and inter-batch precision. The intra-batch and inter-batch precisions must be < 15%, with an accuracy ranging between 85% and 115%.

#### 3.6.4. Stability, Dilution Reliability and Residue Verification

Stability was investigated primarily through storage of stock solutions of dalbavancin components and deuterated dalbavancin in −16 °C to −24 °C for 7 days, storage of whole blood samples at room temperature for 12 h, storage of plasma samples in ice for 4 h, storage of plasma samples following treatment in an automatic sampler for 10 h, storage of plasma samples following treatment at room temperature for 4 h, three freeze-thaw cycles, and storage at −16 °C to −24 °C for 28 days.

During the detection of some plasma samples, the concentrations of B_0_, B_1_, A_1_, and B_2_ samples were beyond the standard curve range. The samples were diluted and retested. Based on the concentration ranges of the experiment, the dilution factors of B_0_ were set at 50 and 1000, while those of B_1_, A_1_, and B_2_ were all set at 50. The accuracy and precision were required to be within the range of ±15%.

For residue verification, residues were determined by examining the upper limit of standard curves and blank plasma samples. The responses measured in blank samples must be < 20% of the lower limit of quantification, with accuracy and precision within ±15%.

### 3.7. Pharmacokinetic Study

A total of 20 SD rats (weighing approximately 200 g each) were selected and divided into two equal groups, with each group containing 5 male and 5 female rats. Pharmacokinetic experiments were conducted for single-dose and multiple-dose administration of dalbavancin hydrochloride. The route of administration was IV, with an administration volume of 5 mL/kg and a dosage was 40 mg/kg. Blood sampling time points of the single-dose administration group were before administration (0 h) and 5 min, 3 h, 8 h, 24 h, 72 h, 144 h, and 216 h post administration. Blood was collected on day 28 for the multiple-dose administration group, and the time points of blood collection were the same as those of single-dose administration. The blood samples collected were immediately separated using centrifugation at 12,000× *g* for 10 min, and then, the plasma were transferred and stored at −16 °C to −24 °C until analysis.

### 3.8. Data Analysis

Microsoft Office Excel 2007 and IBM SPSS Statistics 19.0 were used for data processing and statistical calculation using two-tailed Student’s *t*-test. Data Analysis System 3.1 software (DAS, Shanghai, China) was used to analyze and calculate *AUC_(_*_0–∞)_, *T*_1/2_, *C*_max_ and other parameters using the non-compartmental model.

## 4. Conclusions

In this study, a UHPLC-MS/MS method, with good selectivity, high degree of separation, and high speed, was established for simultaneous determination of the concentration of five components (A_0_, A_1_, B_0_, B_1_, and B_2_) of dalbavancin in rat plasma. The biological analysis method was qualified based on linearity, precision, and stability validation. The LOQ showed good linearity in the range of 50 to 2500 ng/mL. The novelty of this method lies in its ability to rapidly separate several homologs and isomers. In addition, it is an efficient and sensitive method for the simultaneous analysis of trace active ingredients in biological samples. This method was also applied to the pharmacokinetic study of dalbavancin hydrochloride injected in rats for 28 days of continuous administration. The results of the study may help clarify the pharmacokinetics of individual components of dalbavancin in the body. Through further pharmacodynamical and toxicological studies, the concentration at which dalbavancin is safe and effective can be elucidated and the source of AEs associated with dalbavancin can be determined.

## Figures and Tables

**Table 1:** Comparison of the analytical methods used for the quantification of dalbavancin in plasma samples, both reported in literature and proposed in this study.

Methods	Analytes ^1^	Matrix	LOQs (μg/mL)	Reference
HPLC-MS/MS	B_0_/B_1_	Rat plasma	0.5–50	[23]
HPLC-MS/MS	B_0_/B_1_	Human plasma	1.0–128	[24]
HPLC-MS/MS	B_0_/B_1_	Human plasma	0.5–50/1.0–128	[25]
HPLC-MS/MS	B_0_/B_1_	Human plasma	0.5–400	[26]
HPLC-MS/MS	B_0_/B_1_	Human Plasma	0.5–500	[27,28,29,30]
HPLC-MS	B_0_/B_1_	Human Plasma	0.7–500	[31]
LC-MS/MS	B_0_/B_1_	Human Plasma	0.5–500	[32]
UHPLC-MS/MS	B_0_/B_1_	Rat serum	0.002–0.02	[33]
UHPLC-MS/MS	A_0_, A_1_, B_0_, B_1_, B_2_	Rat plasma	0.05–2.5	Present study

^1^ Components of dalbavancin, B_0_/B_1_ refers to component B_0_ or possibly a mixture of B_0_ and B_1_.

**Table 2:** MS conditions and the retention time of UHPLC of dalbavancin and D8-labeled IS.

Analytes	MS Conditions				Retention Time (min)
	Precursor Ion (*m/z*) ^1^	Product Ion (*m/z*) ^2^	Cone Voltage (V)	Collision Energy (eV)	
A_0_	902.19	84.15	54	52	3.20
A_1_	902.25	84.16	54	52	3.42
B_0_	909.21	83.91	64	50	4.69
B_1_	909.29	84.15	64	50	5.03
B_2_	916.25	84.17	15	58	4.92
IS	913.13	158.21	64	48	4.69

^1^ [M+2H]^2+^, ^2^ [M+H]^+^.

**Table 3:** The linear range, limit of detection (LOD), lower limit of quantification (LLOQ), and regression equation of the developed UHPLC-MS/MS method.

Analytes	Linear Range (ng/mL)	LODs (ng/mL)	LLOQs (ng/mL)	Regression Equation	*R*²
A_0_	50–2500	10	50	y = 0.0033x + 0.0110	0.9997
A_1_	50–2500	5	50	y = 0.0031x + 0.0183	0.9999
B_0_	50–2500	5	50	y = 0.0038x + 0.0246	0.9998
B_1_	50–2500	10	50	y = 0.0038x + 0.0168	0.9999
B_2_	50–2500	10	50	y = 0.0037x − 0.0228	0.9990

**Table 4:** Intra-batch precision and accuracy of five homologs of dalbavancin in rat plasma (*n* = 6).

Compound	Concentration (ng/mL)	Precision (RSD, %)		Accuracy (RE, %)	
		Inter-Assay	Intra-Assay	Inter-Assay	Intra-Assay
A_0_	50	2.38–5.96	5.49	98.8–107.6	103.8
	150	2.04–4.42	4.97	96.8−105.9	102.1
	500	1.86–2.74	2.62	98.9−102.1	100.9
	2000	1.48–5.99	4.13	102.6–105.1	103.9
A_1_	50	5.78–7.72	6.36	96.5–106.8	100.9
	150	1.42–2.53	6.10	92.4–105.9	99.8
	500	0.97–2.88	4.77	92.2–101.4	98.1
	2000	1.27–4.74	4.86	95.1–103.3	99.8
B_0_	50	5.41–7.51	6.51	97.4–106.1	102.8
	150	0.98–4.00	4.08	99.0–107.3	103.2
	500	1.45–4.98	3.08	102.4–102.8	102.6
	2000	0.72–3.57	3.12	103.3–108.0	104.9
B_1_	50	3.04–7.32	5.91	98.5–107.5	103.5
	150	3.54–5.49	6.78	97.8–109.9	104.4
	500	1.43–9.84	6.60	105.0–109.1	106.7
	2000	1.79−5.87	3.68	104.4–106.9	105.3
B_2_	50	5.47–6.66	6.15	98.1–105.0	102.2
	150	1.34–3.60	4.40	98.8–108.0	103.1
	500	1.53–3.72	2.90	99.3–102.8	100.8
	2000	0.47–5.52	3.37	100.5–103.6	101.8

**Table 5:** Pharmacokinetic parameters of various dalbavancin components in plasma following IV administration of 40 mg/kg dalbavancin hydrochloride in rats (mean ± SD, *n* = 10).

Stage	Parameters	Components				
		A_0_	A_1_	B_0_	B_1_	B_2_
D_1_	*AUC*_(0–t)_ (μg/mL·h)	10.5 ± 2.6	155.4 ± 79.9	5776.5 ± 2593.2	688.2 ± 272.2	163.7 ± 79.5
	*AUC*_(0–∞)_ (μg/mL·h)	11.8 ± 2.6	173.1 ± 65.6	6566.7 ± 1820.3	756.2 ± 191.4	187.2 ± 61.2
	*T*_1/2_ (h)	6.5 ± 0.6	27.5 ± 11.4	74.6 ± 45.9	29.1 ± 1.1	87.3 ± 68.9
	*C*_max_ (μg/mL)	1.8 ± 0.2	14.8 ± 6.1	437.3 ± 160.3	42.9 ± 18.9	23.4 ± 10.4
	*MRT*_(0–t)_ (h)	5.6 ± 0.3	17.0 ± 3.8	23.0 ± 0.9	32.3 ± 1.5	16.2 ± 2.4
	*V*_z_ (L/kg)	32.9 ± 5.7	9.4 ± 3.3	0.7 ± 0.5	2.3 ± 0.4	28.0 ± 23.0
D_28_	*AUC*_(0–t)_ (μg/mL·h)	34.0 ± 4.5 ***	524.7 ± 32.8 ***	15,781.3 ± 1980.4 ***	3593.7 ± 6,02.1 ***	482.4 ± 35.2 ***
	*AUC*_(0–∞)_ (μg/mL·h)	49.5 ± 8.6 ***	631.0 ± 41.8 ***	18,543.8 ± 2839.6 ***	4333.9 ± 848.9 ***	646.0 ± 53.3 ***
	*T*_1/2_ (h)	1,67.1 ± 32.0 ***	109.5 ± 10.6 ***	88.8 ± 34.5	87.0 ± 27.5 ***	152.8 ± 43.0 *
	*C*_0_ (μg/mL)	0.1 ± 0.0	6.3 ± 0.7	132.6 ± 15.7	37.1 ± 4.1	3.9 ± 0.5
	*C*_max_ (μg/mL)	1.2 ± 0.2 ***	21.1 ± 1.6 ***	571.5 ± 67.4 *	84.3 ± 7.4 ***	28.0 ± 2.6
	*MRT*_(0–t)_ (h)	60.7 ± 2.7 ***	53.0 ± 5.2 ***	62.0 ± 3.7 ***	68.6 ± 8.2 ***	55.7 ± 4.7 ***
	*V*_z_ (L/kg)	196.0 ± 27.3 ***	10.1 ± 1.3	0.3 ± 0.1 *	1.2 ± 0.4 ***	13.5 ± 3.4

* *p* < 0.05, *** *p* < 0.001, when compared to day 1. Abbreviations: *AUC*_(0–t)_, the area under the plasma drug concentration-time curve during the time period from 0 to 216 h, the final time point sampled; *AUC*_(0–∞)_, the area under the plasma drug concentration-time curve during the time period from 0 to infinity; *T*_1/2,_ the time taken for half the initial drug concentration to be eliminated; *C*_max_, the maximum drug concentration; *MRT*_(0–t)_, mean residence time; *V*_z_, apparent volume of distribution; *CL*_z_, Plasma clearance; *C*_0_, the average value of the plasma concentrations before administration. D_1_, D_28_, Days 1, 28 following administration.

## References

[1] Lee A.S., de Lencastre H., Garau J., Kluytmans J., Malhotra-Kumar S., Peschel A., Harbarth S. (2018). Methicillin-Resistant Staphylococcus aureus. Nat. Rev. Dis. Primers.

[2] Holmes N.E., Tong S.Y.C., Davis J.S., van Hal S.J. (2015). Treatment of methicillin-resistant Staphylococcus aureus: Vancomycin and beyond. Semin. Respir. Crit. Care Med..

[3] Shariati A., Dadashi M., Chegini Z., van Belkum A., Mirzaii M., Khoramrooz S.S., Darban-Sarokhalil D. (2020). The global prevalence of daptomycin, tigecycline, quinupristin/dalfopristin, and linezolid-resistant staphylococcus aureus and coagulase-negative staphylococci strains: A systematic review and meta-analysis. Antimicrob. Resist. Infect. Control..

[4] Kim A., Kuti J.L., Nicolau D.P. (2007). Review of dalbavancin, a novel semisynthetic lipoglycopeptide. Expert. Opin. Investig. Drugs.

[5] Soriano A., Rossolini G.M., Pea F. (2020). The role of dalbavancin in the treatment of acute bacterial skin and skin structure infections (ABSSSIs). Expert. Rev. Anti. Infect. Ther..

[6] Pfaller M.A., Mendes R.E., Duncan L.R., Flamm R.K., Sader H.S. (2018). Activity of dalbavancin and comparator agents against Gram-positive cocci from clinical infections in the USA and Europe 2015–16. J. Antimicrob. Chemother..

[7] Goldstein B.P., Selva E., Gastaldo L., Berti M., Pallanza R., Ripamonti F., Ferrari P., Denaro M., Arioli V., Cassani G. (1987). A40926, a new glycopeptide antibiotic with anti-Neisseria activity. Antimicrob. Agents Chemother..

[8] Candiani G., Abbondi M., Borgonovi M., Romanò G., Parenti F. (1999). In-Vitro and in-vivo antibacterial activity of BI 397, a new semi-synthetic glycopeptide antibiotic. J. Antimicrob. Chemother..

[9] Dash R.P., Babu R.J., Srinivas N.R. (2017). Review of the pharmacokinetics of dalbavancin, a recently approved lipoglycopeptide antibiotic. Infect. Dis..

[10] Gustinetti G., Cangemi G., Bandettini R., Castagnola E. (2018). Pharmacokinetic/Pharmacodynamic parameters for treatment optimization of infection due to antibiotic resistant bacteria: A summary for practical purposes in children and adults. J. Chemother..

[11] Galluzzo M., D’Adamio S., Bianchi L., Talamonti M. (2018). Pharmacokinetic drug evaluation of dalbavancin for the treatment of skin infections. Expert. Opin. Drug Metab. Toxicol..

[12] Dunne M.W., Talbot G.H., Boucher H.W., Wilcox M., Puttagunta S. (2016). Safety of dalbavancin in the treatment of skin and skin structure infections: A pooled analysis of randomized, comparative studies. Int. J. Antimicrob. Agents Drug Saf..

[13] Rimkus G.G., Huth T., Harms D. (2020). Screening of stereoisomeric chloramphenicol residues in honey by ELISA and CHARM^®^II test—The potential risk of systematically false-compliant (false negative) results. Food Addit. Contam. Part A Chem. Anal. Control Expo. Risk Assess..

[14] Aga D.S., O’Connor S., Ensley S., Payero J.O., Snow D., Tarkalson D. (2005). Determination of the persistence of tetracycline antibiotics and their degradates in manure-amended soil using enzyme-linked immunosorbent assay and liquid chromatography-mass spectrometry. J. Agric. Food Chem..

[15] Huang L., Yin F., Pan Y., Chen D., Li J., Wan D., Liu Z., Yuan Z. (2015). Metabolism, distribution, and elimination of mequindox in pigs, chickens, and rats. J. Agric. Food Chem..

[16] Hakk H., Shappell N.W., Lupton S.J., Shelver W.L., Fanaselle W., Oryang D., Yeung C.Y., Hoelzer K., Ma Y., Gaalswyk D. (2016). Distribution of animal drugs between skim milk and milk fat fractions in spiked whole milk: Understanding the potential impact on commercial milk products. J. Agric. Food Chem..

[17] Harvanová M., Gondová T. (2019). New enantioselective LC method development and validation for the assay of modafinil. J. Chromatogr. Sci..

[18] D’Orazio G., Fanali C., Gentili A., Tagliaro F., Fanali S. (2019). Nano-Liquid chromatography for enantiomers separation of baclofen by using vancomycin silica stationary phase. J. Chromatogr. A.

[19] Xu Y., Wang D., Tang L., Wang J. (2019). Separation and characterization of unknown impurities and isomers in cefminox sodium and study of the forming mechanisms of impurities by liquid chromatography coupled with ion trap/time-of-flight mass spectrometry. J. Chromatogr. Sci..

[20] Rashid A., Mazhar S.H., Zeng Q., Kiki C., Yu C.P., Sun Q. (2020). Simultaneous analysis of multiclass antibiotic residues in complex environmental matrices by liquid chromatography with tandem quadrupole mass spectrometry. J. Chromatogr. B Anal. Technol. Biomed. Life Sci..

[21] Xiao X., Wu J., Li Z., Jia L. (2019). Enantioseparation and sensitive analysis of ofloxacin by poly(3,4-dihydroxyphenylalanine) functionalized magnetic nanoparticles-based solid phase extraction in combination with on-line concentration capillary electrophoresis. J. Chromatogr. A.

[22] Greño M., Castro-Puyana M., García M.Á., Marina M.L. (2018). Analysis of antibiotics by CE and CEC and their use as chiral selectors: An update. Electrophoresis.

[23] Cavaleri M., Riva S., Valagussa A., Guanci M., Colombo L., Dowell J., Stogniew M. (2005). Pharmacokinetics and excretion of dalbavancin in the rat. J. Antimicrob. Chemother..

[24] Nicolau D.P., Sun H.K., Seltzer E., Buckwalter M., Dowell J.A. (2007). Pharmacokinetics of dalbavancin in plasma and skin blister fluid. J. Antimicrob. Chemother..

[25] Buckwalter M. (2005). Population pharmacokinetic analysis of dalbavancin, a novel lipoglycopeptide. J. Clin. Pharmacol..

[26] Bradley J.S., Puttagunta S., Rubino C.M., Blumer J.L., Dunne M., Sullivan J.E. (2015). Pharmacokinetics, safety and tolerability of single dose dalbavancin in children 12–17 years of age. Pediatr. Infect. Dis. J..

[27] Alebic-Kolbah T., Demers R., Cojocaru L. (2011). Dalbavancin: Quantification in human plasma and urine by a new improved high performance liquid chromatography-tandem mass spectrometry method. J. Chromatogr. B Anal. Technol. Biomed. Life Sci..

[28] Carrothers T.J., Chittenden J.T., Critchley I. (2020). Dalbavancin population pharmacokinetic modeling and target attainment analysis. Clin. Pharm. Drug Dev..

[29] Gonzalez D., Bradley J.S., Blumer J., Yogev R., Watt K.M., James L.P., Palazzi D.L., Bhatt-Mehta V., Sullivan J.E., Zhang L. (2017). Dalbavancin pharmacokinetics and safety in children 3 months to 11 years of age. Pediatr. Infect. Dis. J..

[30] Van Matre E.T., Teitelbaum I., Kiser T.H. (2020). Intravenous and intraperitoneal pharmacokinetics of dalbavancin in peritoneal dialysis patients. Antimicrob. Agents Chemother..

[31] Corona A., Agarossi A., Veronese A., Cattaneo D., D’Avolio A. (2020). Therapeutic drug monitoring of dalbavancin treatment in severe necrotizing fasciitis in 3 critically ill patients a grand round. Ther. Drug Monit..

[32] Rappo U., Dunne M.W., Puttagunta S., Baldassarre J.S., Su S.F., Desai-Krieger D., Inoue M. (2019). Epithelial lining fluid and plasma concentrations of dalbavancin in healthy adults after a single 1500-milligram infusione. Antimicrob. Agents Chemother..

[33] Deng F.F., Yu H., Pan X.H., Hu G.Y., Wang Q.Q., Peng R.F., Tan L., Yang Z.C. (2018). Ultra-High performance liquid chromatography tandem mass spectrometry for the determination of five glycopeptide antibiotics in food and biological samples using solid-phase extraction. J. Chromatogr. A.

[34] Desmons A., Thioulouse E., Hautem J.Y., Saintier A., Baudin B., Lamazière A., Netter C., Moussa F. (2020). Direct liquid chromatography tandem mass spectrometry analysis of amino acids in human plasma. J. Chromatogr. A.

[35] Lu X., Zhang L., Sun Q., Song G., Huang J. (2019). Extraction, identification and structure-activity relationship of antioxidant peptides from sesame (*Sesamum indicum* L.) protein hydrolysate. Food Res. Int..

[36] Li Y., Sun M., Mao X., Li J., Sumarah M.W., You Y., Wang Y. (2019). Tracing major metabolites of quinoxaline-1,4-dioxides in abalone with high-performance liquid chromatography tandem positive-mode electrospray ionization mass spectrometry. J. Sci. Food Agric..

[37] Cheng C., Liu S., Xiao D., Hollembaek J., Yao L., Lin J., Hansel S. (2010). LC-MS/MS method development and validation for the determination of polymyxins and vancomycin in rat plasma. J. Chromatogr. B.

[38] Bioanalytical Method Validation Guidance for Industry, U.S. Department of Health and Human Services, Food and Drug Administration, Center for Drug Evaluation and Research (CDER), Center for Veterinary Medicine (CVM), May 2018, Food and Drug Administration Guidance for Industry: Bioanalytical Method Validation. https://www.fda.gov/regulatory-information/search-fda-guidance-documents/bioanalytical-method-validation-guidance-industry.

[39] European Commission (2006). [EC] (2006b), Commission regulation (EC) no 401/2006 of 23 February 2006 laying down the methods of sampling and analysis for the official control of the levels of mycotoxins in foodstuffs. Off. J. Eur. Union.

[40] European Commission (2002). 2002/657/EC, Commission Decision of 12 August 2002 implementing Council directive 96/23/EC concerning the performance of analytical methods and the interpretation of results. Off. J. Eur. Commun..

